# Functionalized polymers: synthesis and properties

**DOI:** 10.3762/bjoc.6.55

**Published:** 2010-06-01

**Authors:** Helmut Ritter

**Affiliations:** 1 Institute of Organic Chemistry and Macromolecular Chemistry II, Heinrich-Heine-University of Düsseldorf, Universitätsstraße 1, D-40225 Düsseldorf, Germany

Materials that are used in everyday life have an immense impact on the development of the human society. During the Stone Age and Iron Age epochs, humans widely used stone or iron to make tools. In the present age, there is no question that polymers and plastics dominate our rapidly developing daily needs and show enormous potential for the development of new technologies. Constructive materials, for instance, are preferentially made of standard polymeric materials such as polyolefins, polyesters or polyamides. It is therefore obvious that the future of polymer chemistry will be influenced by the elaboration of new functional polymers. At the beginning of the application of synthetic materials, naturally occurring polymers as cellulose or polyisoprene were simply modified, for example by esterification or cross-linking to obtain the desired properties. Hermann Staudinger, for instance, chemically modified starch to prove the existence of high molecular weight substances. Nowadays, the development of various functional polymers is becoming increasingly important in specific areas of application.

In terms of technical applications, functional polymers are important, for example in the fields of optics, electronics or catalysis. They are also widely used for analytical devices, (e.g. columns for chromatography), for membranes and in the solid phase synthesis of peptides and oligonucleotides.

Focusing on the constantly developing medical field, only specifically elaborated functional polymer materials can fulfill the specific challenges required of them for use as, for example, surgical sutures, dental fillings, wound dressings, bone cements or hollow fibers for dialysis. Typical examples of such materials include hydrogels and stimulus-responsive polymers which are also the basis of ophthalmic surgery.

Thus, our aim in this Thematic Series is to bring together a broad spectrum of reports in the field of functionalized polymers to update the “state of the art” of knowledge in this field. It is therefore a great pleasure to serve as an editor for this series of articles.

Helmut Ritter

Düsseldorf, May 2010

